# Polymorphisms of Proinflammatory Cytokines in Relation to APOE Epsilon 4 and Risk of Alzheimer’s Disease in the Lithuanian Population

**DOI:** 10.3390/medicina55100689

**Published:** 2019-10-15

**Authors:** Greta Pšemeneckienė, Kęstutis Petrikonis, Daiva Rastenytė

**Affiliations:** Department of Neurology, Medical Academy, Lithuanian University of Health Sciences, LT-50161 Kaunas, Lithuania; kestutis.petrikonis@lsmuni.lt (K.P.); daiva.rastenyte@lsmuni.lt (D.R.)

**Keywords:** TNF alpha, interleukin, polymorphism, APOE, Alzheimer’s disease

## Abstract

*Background and objective:* Neuroinflammation is one of the pathological pathways of Alzheimer’s disease (AD), mediating the progression of neurodegeneration. Polymorphisms of proinflammatory cytokines have been linked to increased AD risk. Identification of certain combinations of polymorphisms could help predict disease in its preclinical stage. The aim of the study was to evaluate differences in the prevalence of TNF*α* –850T (rs1799724), IL1A –889T (rs1800587), and IL6 –174C (rs1800795, Intron type) polymorphisms between AD patients and healthy controls (HC) and determine the impact of these SNPs in combination with the APOEε4 allele on AD risk. *Materials and Methods:* The study population is comprised of 107 patients with sporadic AD (AD group) and age- and gender-matched 110 persons without impaired cognitive functions (control group). TNF*α* –850C > T polymorphism was revealed by a PCR and restriction fragment length polymorphism (RFLP) method. Real time PCR was used for IL1A and IL6 SNP genotyping. APOEε genotyping was done via hybridization method. *Results:* The frequencies of TNF*α* –850T, IL1A –889T, IL6 –174C allele and genotype did not differ between the AD and HC groups (*p* > 0.05). IL6 –174C was not in HWE, and it was not analysed further. APOEε4 allele (*p* = 0.001) and 3/4 and 4/4 genotypes (*p* = 0.005) were more prevalent in AD patients. APOEε4 carriage increased the risk of AD (OR 2.65, *p* = 0.001), while TNF*α* –850T and IL1A –889T polymorphisms were not found as significant independent risk factors for AD. The presence of at least one IL1A –889T allele in combination with APOEε4+ was associated with a lower risk of AD (OR 2.24, *p* = 0.047) than the carriage of APOEε4+ alone (OR 2.70, *p* = 0.015). *Conclusions:* No significant differences of TNFα –850, IL1A –889, and IL6 –174 polymorphisms frequencies were found between AD and control groups. In APOEε4 carriers IL1A –889T polymorphism was found to reduce the AD risk determined by APOEε4 alone.

## 1. Introduction

Alzheimer’s disease (AD) is a neurodegenerative multi-etiological disorder, where the amyloidogenic pathway is considered as the cornerstone of pathology. Tau protein-related changes and neuroinflammatory processes also play an important role in the progression of neurodegeneration. Enhancement of microglia activity was stated in vivo studies with AD patients [1], suggesting neuroinflammation as an early event in AD pathogenesis. It has also been noted that the levels of inflammatory cytokines (tumour necrosis factor-alpha (TNF*α*), interleukin 1 (IL1), interleukin 6 (IL6), interleukin 8 (IL8), interleukin 10 (IL10), etc.) differ in AD patients compared with cognitively healthy people both in peripheral blood and cerebrospinal fluid (CSF) [2,3]. Neuroinflammatory processes may produce blood–brain barrier disruption in the early stage of AD pathology [4]. A wide range of genetic cytokine polymorphisms has been reported to be related to AD risk, but most of the data remain controversial or may be different due to specific ethnic DNA features [5].

TNF*α* is one of the most investigated, neurodegeneration-linked proinflammatory factors. TNF*α* as a central mediator regulates different processes in the CNS, including neuronal development, cell survival, synaptic transmission, and neuronal ionic homeostasis [6]. TNF*α* is in close relation to amyloid beta (Aβ), which possibly stimulates Toll-like receptors and TNF*α* expression [7]. On the other hand, stimulation of neuroinflammation and TNF*α* signaling may induce or maintain the production of new pathogenic amyloid beta [6]. Several single-nucleotide polymorphisms (SNPs) of TNFα have been suggested as possible genetic risk factors for AD. The polymorphism in the promoter region (–850) of TNF*α* gene (TNF*α* –850C > T, rs1799724) has been associated with a higher risk of AD and synergistic effect with APOE epsilon 4 allele (APOEε4) [8,9,10]. However, the role of TNF*α* –850T remains doubtful, as studies in other populations failed to confirm the association between AD risk and TNFα –850T polymorphism [5,11].

IL1 is another proinflammatory cytokine associated with AD. It has been reported that IL1 restrains the function of cholinergic systems [12] and promotes the deposition of amyloid beta plaques and the accumulation of neurofibrillary tangles [13]. Polymorphism of the gene encoding IL1 alpha (IL1A) in its 5′ regulatory region IL1A –889 C > T (rs1800587) has been reported to be associated with AD risk in Caucasians [5,14,15], but the results of other studies have been inconsistent [16,17].

IL6 has been suggested as a major proinflammatory cytokine in the CNS being important in the pathogenesis of the main neurodegenerative pathologies such as AD, Parkinson’s disease, multiple sclerosis, Huntington’s disease, and even some psychiatric disorders [18]. Overexpression of IL6 was observed in the brain of AD patients, where it was linked to amyloidogenesis [19,20]. IL6 was found to be elevated in the CSF and blood serum in AD cases [21], and it was demonstrated that IL6 levels could possibly indicate the severity of AD [22]. The protective role of IL6 –174G > C (rs1800795, Intron type) gene promoter polymorphism was observed [23,24,25], while other authors reported contrary results [26].

To our knowledge, there are no studies on the polymorphisms of inflammatory cytokines in the Lithuanian population in the context of dementia and AD risk. As single polymorphisms were not shown to be strongly linked to AD risk, we aimed at determining the associations between AD risk and TNF*α* –850T (rs1799724), IL1A –889T (rs1800587), and IL6 –174C (rs1800795, Intron type) polymorphisms in combination with APOEε4, which is still considered to be the strongest genetic risk factor for sporadic AD [27].

## 2. Materials and Methods

### 2.1. Study Population

The permission to conduct this case-control study was issued by the Kaunas Regional Biomedical Research Ethics Committee (No. BE-2-47, supplement No. P1-BE-2-47/2017).

The study was carried out in the Department of Neurology, Hospital of Lithuanian University of Health Sciences (LSMU) Kauno klinikos (Kaunas, Lithuania). A total of 217 persons aged more than 50 years, who had no major somatic disease (decompensated heart failure, terminal renal or hepatic dysfunction, or active cancer) or no severe mental disorder (psychotic type, severe depression), were included in the study. The AD group consisted of patients diagnosed with possible sporadic AD according to the NINCDS-ADRDA Alzheimer’s Criteria [28]. All of them were screened for thyroid dysfunction and megaloblastic anemia. Exclusion criteria were prominent neurological deficit (severe paresis, ataxia, aphasia, etc.) and evident extrapyramidal signs (tremor, rigidity) or focal lesions in brain imaging (CT scan or MRI). Patients with AD were consulted together with a family member or other accompanying person. The control group (healthy controls, HC) consisted of persons with no cognitive disorder, matched by gender and age, who were under investigation or treated for others, but not a degenerative neurological disorder in the Department of Neurology. AD patients with their caregivers and controls were introduced with the study, received written information, and signed informed consent form.

Each participant completed a questionnaire on general demographic information and risk factors (age, duration of formal education in years, family history, lifestyle, and other diseases and conditions). Neurological status was evaluated by a standard neurological examination. All demented patients (AD group) were consulted by a psychiatrist in order to rule out pseudo dementia. Cognitive status was assessed using the Mini-Mental State Examination (MMSE) and the Blessed dementia scale [29,30]. Early stage AD (E-AD) was defined as mild dementia (MMSE score of 21–24) and minor disturbance of daily activities (Blessed’s dementia scale score of 4–9), middle stage AD (M-AD), as moderate AD-type dementia (MMSE score of 11–20) and moderate daily life dysfunction (Blessed’s dementia scale score of 10–22), and late AD, as severe dementia (MMSE score of 10 or less) and moderate-to-severe daily life disability (Blessed’s scale score of 10 to 28). Patients both with early onset AD (EOAD, diagnosed with AD at the age of <65 years) and late onset AD (LOAD, aged 65 years and more) were included. The peripheral blood sample (5 mL of venous blood in EDTA collection tube) for DNA extraction was drawn by an experienced nurse.

### 2.2. Genotyping

DNA was extracted from peripheral blood lymphocytes by a standard procedure using an automatic DNA extraction kit (QIAmp^®^DNA Blood Mini Kit, Qiagen, Hilden, Germany).

TNF*α* –850C > T (rs1799724) polymorphism was detected by the polymerase chain reaction (PCR) and restriction fragment length polymorphism (RFLP) analysis. TNF*α* –850 SNP genotype was determined by PCR amplification in a total volume of 30 µL, containing HotStart Taq Master Mix (Qiagen, 1000 U), each forward and reverse TNF*α* –850 primers (10 µM), under the following conditions: 1 cycle of 95 °C for 15 min, 35 cycles of 94 °C for 45 s, 60 °C for 30 s, and 72 °C for 45 s, followed by 1 cycle of 72 °C for 5 min. The primers 5′-TCG AGT ATC GGG GAC CCC CCG TT-3′ (underline denotes mismatch) and 5′-CCA GTG TGT GGC CAT ATC TTC TT-3′ (modified from the initially published [31]) were used (from Integrated DNA Technologies, BVBA, Leuven, Belgium). Amplified TNF*α* –850 was digested at 37 °C for 4 h using 500 U HincII (concentration 10 U/µL) (restriction sequence GTT▲AAC), separated on 4% agarose gels at 100 V (60 min) and stained with ethidium bromide to reveal DNA fragments with migration patterns specific for each allele (C allele—105 bp and 23 bp, T allele—128 bp) [32] (FastDigest Hinc II, Thermo Fisher Scientific, Life Technologies Ltd, Paisley, UK).

Genotyping of IL1A –889 (rs1800587) and IL6 –174 (rs1800795, Intron type) SNP’s polymorphism was performed using a real-time PCR method. PCR amplification was done in a total volume of 25 µL, containing Type-it Fast SNP Probe PCR Master Mix (12.5 µL), primer–probe mix (1.25 µL), DNA (20 ng), and RNase-free water, by the following PCR cycling program: 1 cycle of 95 °C for 5 min for initial PCR activation, followed by 40 cycles of 95 °C for 15 s and 60 °C for 30 s (Type-it Fast SNP Probe PCR Kit, Qiagen). After PCR amplification, end-point plate reading was completed on a real-time PCR instrument (Qiagen RotorGene Q, Qiagen, Hilden, Germany) based on fluorescence measurements. The allele detected by VIC was IL1A –889A (T) and IL6 –174C, the allele detected by FAM was IL1A –889G (C) and IL6 –174G. The primers GAT TTT TAC ATA TGA GCC TTC AAT G[A/G]T GTT GCC TGG TTA CTA TTA TTA AAG (IL1A –889), and ACT TTT CCC CCT AGT TGT GTC TTG C[C/G]A TGC TAA AGG ACG TCA CAT TGC ACA (IL6 –174) were used (TaqMan ^®^, ThermoFischer Scientific, Life Technologies Ltd, Paisley, UK).

APOE ε allele was determined via the hybridization method according to the manufacturer’s protocol (GenoType ApoE, ver. 1.0, 2015, Hain Lifescience GmbH, Qiagen, Nehren, Germany).

### 2.3. Statistical Analysis

Data were analyzed using the Statistical Package for the Social Sciences (IBM^®^ SPSS Statistics, IBM, Armonk, NY, USA) version 23.0 software. Sample size was calculated using the formula:

*n* = [(Z_α/2_ + Z_β_)^2^ × {(*p*1 (1 − *p*1) + (*p*2 (1 − *p*2))}]/(*p*1 − *p*2)^2^

where *n* = sample size required in each group, *p*1 = 0.18, *p*2 = 0.35, *p*1 − *p*2 = significant difference = 0.17 (according to expected genotype frequency), Z_α/2_: for level of significance 5% this is 1.96, Z_β_: for 80% power is 0.84. Based on the above formula, the sample size required per group is 102 patients (204 persons in total). The normality of data distribution of continuous variables was tested by the Shapiro-Wilk test. Normally distributed variables (Shapiro-Wilk test *p* < 0.05) were expressed as mean (standard deviation, SD). For variables that were not normally distributed, nonparametric tests were applied and results are expressed as median (IQR). Results are presented as numbers (percentages) for categorical variables. Allele frequencies were tested for the Hardy-Weinberg equilibrium (HWE). Pearson’s chi-square (*χ*^2^) test was used to compare genotype and allele frequencies between AD and HC groups. Binary logistic regression analysis, adjusted for age and gender, was performed to estimate the possible effect of TNF*α* –850T, IL1A –889T, IL6 –174C polymorphisms and combinations of cytokine polymorphisms with APOEε4 on AD risk in the AD patients and controls (development of AD as a dependent variable). Odds ratios (ORs) with 95% confidence intervals (95% CI) were calculated. The level of significance level was set at <0.05.

## 3. Results

A total of 217 persons were included in the study. All the participants were Caucasian, most of them were of Lithuanian origin. AD patients and controls were matched for age and gender (Table 1).

Patients with AD group had lower BMI and fewer years of education, and a family history of dementia was documented more frequently among patients with AD. As expected, inheritance of at least one APOE epsilon 4 allele (APOEε4+) was more prevalent in the AD group (Table 1). APOEε 3/4 and 4/4 genotypes were more frequent in AD patients, while APOEε 3/3 was more prevalent in the controls group (Table 2).

In the AD group, there were 17 patients (15.9%) with early onset AD (EOAD) and 90 patients (84.1%) with late onset AD (LOAD). Of the AD patients, 29% (*n* = 31) had mild AD, 62.6% (*n* = 67) had moderate AD, and 8.4% (*n* = 9) had severe AD.

The distribution of TNF*α* –850 and IL1A –889 alleles and genotypes followed the Hardy–Weinberg equilibrium (AD group HWE: TNF*α* — *p* = 0.69, IL1A — *p* = 0.97, HC group HWE: TNF*α* — *p* = 0.83, IL1A — *p* = 0.61). The distribution of IL6 –174 did not meet HWE both in AD patients and controls (*p* < 0.05), thus it was not analysed further than evaluation of allele and genotype frequencies. The very homogenous ethnic origin of subjects in our study possibly could explain that, because IL6 –174 SNP genotype and allele distribution varies considerably according to the population ethnicity [33]. No significant difference was found in the distribution of cytokine genotype and allele between the AD and control groups (Table 3).

There were no significant differences in the frequencies of genotypes among subjects who had at least one allele of the expected polymorphism (dominant genotype model: TNF*α* –850(TT + CT), IL1A –889(TT + TC)). Moreover, there were no significant differences comparing the distribution of polymorphisms between the patients with EOAD (age at collection: mean 61.59 ± 4.23, median 63.0, min. 54, max. 68 years) and LOAD (age at collection: mean 75.99 ± 5.11, median 75.0, min. 66, max. 88 years, age EOAD *vs* LOAD *p* = 0.001) (Table 4).

Binary logistic regression analysis, adjusted for age and gender (independent variables), showed that the presence of APOEε4 allele was associated with more than a 2-fold higher risk of AD (OR 2.65, 95% CI 1.49–4.70, *p* = 0.001). TNF*α* –850T and IL1A –889T polymorphisms were not associated with AD risk (*p* > 0.05) (Table 5), and entering APOEε4 as an additional covariate (model^2^ with age, gender, and APOE as independent variables) into logistic regression analysis did not have any significant impact for associations (Table 5).

The presence of at least one IL1A –889T allele in combination with APOEε4+ was associated with a lower risk of AD (OR 2.24, *p* = 0.047) than the carriage of APOEε4+ alone (OR 2.70, *p* = 0.015) (Table 6).

Multivariate logistic regression analysis (backward method) revealed that APOEε4 allele was associated with higher AD risk (OR 2.80, 95% CI 1.49–5.26, *p* = 0.001), while longer duration of education (OR 0.87, 95% CI 0.80–0.94, *p* = 0.001) and higher body mass index (OR 0.91, 95% CI 0.84–0.97, *p* = 0.006) had a protective effect.

## 4. Discussion

Searching for certain combinations of AD-related polymorphisms has been a challenge for many researchers. The main reason forcing investigators to put in the effort is the ambition to predict disease in its early stages before cognitive decline is observed. This could lead to timely treatment and reduce the burden of Alzheimer’s dementia. In this study, we investigated the relation of proinflammatory cytokine polymorphisms to AD risk in association with the carriage of APOE ε4 allele. This study is the first one to evaluate the impact of TNF*α*, IL1, and IL6 polymorphisms on AD risk in the Lithuanian population. Our results on the prevalence of APOEε4 allele correspond to other published data showing that the prevalence of this allele ranges from 40% to 50% among AD cases and support the recognition of APOE as the main genetic risk factor of AD susceptibility [27]. We found the epsilon 4 allele to be almost 2-fold more frequent in patients with AD, and the prevalence of APOEε genotypes 3/4, 4/4 was higher in this group as well. It has been reported that APOEε4 is associated with sporadic and familial AD [34,35,36], and the carriage of ε4 allele may determine a higher risk to develop AD at younger age [37]. Patients with both late and early onset AD were included in our study, but our data do not let us make explicit conclusions on the influence of the APOE polymorphism according to the age of disease onset. However, clinical observations suggest that in patients with at least one ε4 allele, cognitive functions tend to deteriorate in a more aggressive way and this could be a focus for further investigations. We also found that APOE ε2 allele and ε2/2, 2/3 genotypes were more prevalent in healthy controls, and these findings are in line with the results of studies in other populations [38,39]. Thus, APOEε2 could be an indirect protective biomarker of AD development, useful to know in the clinical settings.

The results of TNF*α* –850T polymorphism revealed some tendency, comparing the distribution of TT homozygotes together with CT heterozygotes (dominant model) between AD and healthy control groups. We also observed that the combination of APOEε4+ and TNF*α* –850T could increase the risk of AD reasonably, and it just slightly miss significance (*p* = 0.053), possibly because of the sample size. This statement would be in line with data reported by other authors stating that TNF*α* –850T allele with APOEε4 has a synergistic effect on greater AD risk in European populations [8,9,10]. The Australian study on patients with late onset AD also noted that TNF*α* –850T contributed to AD risk independently of APOEε4 carriage [32]. Still data are inconsistent to make definite conclusions as other studies in different populations have reported opposite results and have not proven the association between AD risk and TNF*α* –850T polymorphism [11,40]. The inconsistent findings probably could be explained by ethnic differences and diversity of study design or certain peculiarities of patient involvement in the research (for example, different diagnostic criteria were used, patients only with late onset or not specified sporadic AD were involved, or only cases with AD verified by pathological findings).

The current study showed contradictory results on the polymorphisms of both IL1A –889T and IL6 –174C. The prevalence of alleles (IL1A –889 C and T, IL6 –174C and G) was similar to that reported in other Europe populations [33]. The distribution of genotypes and alleles of those SNPs had no significant difference in AD versus controls (chi square test, *p* > 0.05). IL1A –889T had no significant impact on AD risk when evaluating separately. However, in APOEε4 carriers, IL1A polymorphism had a significant influence on AD risk. Polymorphism of IL1A –889T (at least one T allele) was associated with AD risk in APOEε4+ cases, but the effect was the reverse, antagonistic to APOE, suggesting IL1A –889T could lower the AD risk. This is in disagreement with the conclusions of a recent meta-analysis by Dong et al. [41], where the significant correlation between IL1A polymorphism and AD risk was stated and T allele considered to be a factor for AD susceptibility. Of course, we must interpret these our results with caution, because in the combination with IL1A -889T, inheritance of APOEε4, but not the 1L1A polymorphism, still had a decisive effect on AD risk. On the other hand, different authors also declare negative results according to IL1A polymorphism and AD risk [42,43,44]. Specific combinations of polymorphisms due to the ethnicity of patients could possibly explain the discrepancy of the results, and the age of disease onset could be considered too. Rebeck reported that IL1A –889TT genotype was associated with an earlier age of AD onset and increased risk of EOAD [45].

The impact of the IL6 –174C polymorphism on AD risk was not analysed in our study because of its deviation from Hardy-Weinberg equilibrium. In previous studies, the protective role of the CC genotype in AD manifestation has been documented [23,24]. Although in several studies, the role of IL6 polymorphism has also been argued, and the evident association has not been established [26,46]. Certainly, we have to keep in mind that the small sample size of our study might impact the results, and there is the possibility of false negative results in the differences between genotype and allele distributions. On the other hand, there is the possibility of false positive results in estimation of TNFα, IL1A polymorphisms and APOE interaction, as no correction for multiple testing was applied. Further studies are needed to confirm or contradict our observations. Despite inconsistent data on associations between the polymorphisms of proinflammatory factors and AD risk, associations with blood and CSF findings [47,48], possible specific AD genetic profiles [49] make further investigations worthy, expecting new biomarkers to be suggestive of Alzheimer’s dementia. Precise selection of study cases targeted on age of disease onset, BMI, and education could help disclose beneficial information on AD risk. Our study revealed the protective role of longer duration of education, and this in agreement with the results of other investigators [50,51]. Moreover, we found a significant impact of BMI on AD risk. Patients with lower BMI were at greater risk of AD, and from the first glance it looks conflicting with the concept of obesity as a risk factor for dementia [52,53,54]. It was suggested that metabolism changes determined by obesity in middle age of lifespan could be in relation to neuroinflammation and structural brain grey matter changes [52,53]. However, a recent review on 39 cohort studies with more than 1.3 million individuals indicates that higher BMI is associated with an elevated risk of dementia only if it would be evaluated two decades or more before cognitive disturbance. Lower BMI could reflect the higher dementia risk assessing BMI more close (less than 10 years) to the clinical cognitive decline [54]. The main limitation of the study was a quite small sample size. More valuable insights could be expected if the study data could be interpreted in the context with other biomarkers of AD such as CSF or blood amyloid and tau protein level profile or biomarkers imaging (Abeta or Tau PET) findings.

## 5. Conclusions

In conclusion, we found no significant differences in the frequencies of polymorphisms of proinflammatory cytokines TNF*α* –850T, IL1A –889T, and IL6 –174C between AD patients and persons with intact cognitive functions. TNF*α* –850T, IL1A –889T polymorphisms had no significant impact on AD risk, thus according to our data, TNFα –850T and IL1A – 889Tcannot be considered as independent risk factors for developing AD. The carriage of the APOEε4 allele was associated with greater AD risk. Longer duration of education and greater BMI appeared to have a protective role in AD. In APOEε4-positive persons, the inheritance of IL1A –889T polymorphism (at least one T allele) was found to reduce the AD risk determined by the possession of APOEε4 alone.

## Figures and Tables

**Table 1:** Characteristics of the study population.

Characteristic	AD (*n* = 107)	HC (*n* = 110)	*p*
Age, mean (SD), years	73.77 (7.3)	73.03 (7.5)	0.462
Gender, *n* (%)			
Male	35 (32.7)	37 (33.6)	0.885
Female	72 (67.3)	73 (66.4)	
APOEε4+, *n* (%)	51 (48.6)	29 (26.6)	0.001
MMSE, median (IQR), score	19 (15–27)	28 (28–29.25)	<0.001
Education, median (IQR), years	12 (8–15)	14 (11–16)	0.004
BMI, median (IQR), kg/m^2^	25.45 (23.18–28.93)	27.36 (24.29–30.9)	0.009
Family history of dementia, *n* (%)	36 (33.6)	16 (14.5)	0.001

AD—Alzheimer’s disease, HC—healthy controls, MMSE—Mini-Mental State Examination, BMI—body mass index, APOEε4+—subjects with at least one APOE epsilon 4 allele. Normally distributed data were compared with the Student’s *t* test, non-normally distributed continuous data, with the Mann–Whitney *U* test, and categorical data, with Pearson’s chi-square test.

**Table 2:** Distribution of APOE ε allele and genotype between AD patients and controls.

APOE	AD (*n* = 105)	HC (*n* = 109)	*p*
Genotype, *n* (%)			
2/2	1 (1.0)	2 (1.8)	0.005
2/3	7 (6.7)	15 (13.8)	
3/3	46 (43.8)	63 (57.8)	
3/4	44 (41.9)	25 (22.9)	
4/4	4 (3.8)	0 (0)	
2/4	3 (2.9)	4 (3.7)	
Alleles, *n* (%)			
2	12 (5.7)	23 (10.6)	
3	143 (68.1)	166 (76.1)	0.001
4	55 (26.2)	29 (13.3)	

AD—Alzheimer’s disease group, HC—healthy controls. Values were compared with Pearson’s chi-square test.

**Table 3:** Distribution of TNF*α* –850, IL1A –889 and IL6 –174 alleles and genotypes between AD patients and controls.

SNP	Genotype/Alleles	AD	HC	*p*
TNF*α* –850		*n* = 98	*n* = 106	
	Genotype, *n* (%)			
	CC	81 (82.7)	91 (85.8)	0.522
	CT	16 (16.3)	15 (14.2)	
	TT	1 (1.0)	0 (0.0)	
	CT + TT	17 (17.3)	15 (14.2)	0.531
	Alleles, *n* (%)			
	C	178 (90.8)	197 (92.9)	0.471 *
	T	18 (9.2)	15 (7.1)	
ILA –889		*n* = 107	*n* = 109	
	Genotype, *n* (%)			
	CC	59 (55.1)	56 (51.4)	0.761
	CT	41 (38.3)	47 (43.1)	
	TT	7 (6.5)	6 (5.5)	
	CT + TT	48 (44.9)	53 (48.6)	0.579
	Alleles, *n* (%)			
	C	159 (74.3)	159 (72.9)	0.748
	T	55 (25.7)	59 (27.1)	
IL6 –174		*n* = 107	*n* = 109	
	Genotype, n (%)			
	CC	26 (24.3)	27 (24.8)	0.798
	CG	66 (61.7)	70 (64.2)	
	GG	15 (14.0)	12 (11.0)	
	Alleles, *n* (%)			
	C	118 (55.1)	124 (56.9)	0.716
	G	96 (44.9)	94 (43.1)	

AD—Alzheimer’s disease group, HC—healthy controls. Genotype and allele frequencies were compared using the chi-square test and Fisher’s exact test (*).

**Table 4:** Distribution of TNF*α* –850 and IL1A –889 alleles and genotypes between EOAD and LOAD patients and controls.

SNP	Genotype/Alleles	EOAD	LOAD	HC	*p*
TNF*α* –850		*n* = 17	*n* = 81	*n* = 106	
	Genotype, *n* (%)				
	CC	16 (94.1)	65 (80.2)	91 (85.8)	0.413 ^1^
	CT	1 (5.9)	15 (18.5)	15 (14.2)	0.696 ^2^
	TT	0 (0.0)	1 (1.2)	0 (0.0)	0.363 ^3^
	Alleles, *n* (%)				
	C	33 (97.1)	145 (89.5)	197 (92.9)	0.322
	T	1(2.9)	17(10.5)	15 (7.1)	
ILA –889		*n* = 17	*n* = 90	*n* = 109	
	Genotype, *n* (%)				
	CC	9 (52.9)	50 (55.6)	56 (51.4)	0.418 ^1^
	CT	8 (47.1)	33 (36.6)	47 (43.1)	0.608 ^2^
	TT	0 (0.0)	7 (7.8)	6 (5.5)	0.588 ^3^
	Alleles, *n* (%)				
	C	26 (76.5)	133 (73.9)	159 (72.9)	0.833
	T	8 (23.5)	47 (26.1)	59 (27.1)	

EOAD—early onset Alzheimer’s disease group, LOAD—late onset Alzheimer’s disease group, HC—healthy controls. Genotype and allele frequencies were compared using the chi-square test. *p—*EOAD vs. LOAD (allele), *p*^1^—EOAD vs. LOAD, *p*^2^—EOAD vs. HC, *p*^3^—LOAD vs. HC (genotype).

**Table 5:** Logistic regression analysis of TNF*α* –850C > T and IL1A –889C > T SNPs in Alzheimer’s disease and control groups.

SNPs	Model		β	SE	Wald Statistics	OR (95% CI)	*p*
TNFα –850C > T	CC	Ref.					
	CT+TT	dominant^1^	0.23	0.39	0.37	1.26 (0.59–2.70)	0.545
		dominant^2^	0.33	0.41	0.66	1.39 (0.62–3.09)	0.417
IL1A –889C > T	CC	Ref.					
	CT+TT	dominant^1^	−0.15	0.27	0.3	0.86 (0.50–1.47)	0.585
		dominant^2^	−0.14	0.28	0.26	0.87 (0.50–1.51)	0.61
	CC+CT	Ref.					
	TT	recessive^1^	0.19	0.58	0.11	1.21 (0.39–3.73)	0.744
		recessive^2^	0.21	0.59	0.13	1.23 (0.39–3.93)	0.724

SNP—single nucleotide polymorphism, dominant^1^, recessive^1^—model for binary logistic regression, adjusted for age and gender, dominant^2^, recessive^2^—model for binary logistic regression, adjusted for age, gender and carriage of at least one APOE ε4 allele, Ref.—reference group, β—coefficients beta, SE—standard error, OR—odds ratio, CI—confidence interval.

**Table 6:** Logistic regression analysis of the interaction between APOEε4 allele and polymorphisms of TNF*α* and IL1A in Alzheimer’s disease and control groups.

Genotypes		Frequencies		Logistic Regression		
		HC	AD	β; SE; Wald	Odds Ratio (95% CI)	*p* Value
		*n* (%)	*n* (%)			
APOEε4	TNF*α* –850T	*n* = 105	*n* = 96			
						0.005
–	–	66 (62.9)	39 (40.6)		Ref.	
–	+	13 (12.4)	10 (10.4)	0.26; 0.47; 0.32	1.30 (0.52–3.25)	0.572
+	–	24 (22.9)	41 (42.7)	1.06; 0.33; 10.55	2.89 (1.52–5.49)	0.001
+	+	2 (1.9)	6 (6.3)	1.63; 0.84; 3.73	5.08 (0.98–26.40)	0.053
APOEε4	IL1A –889T	*n* = 109	*n* = 105			
						0.012
–	–	42 (38.53)	30 (28.57)		Ref.	
–	+	38 (34.86)	24 (22.86)	−0.12; 0.35; 0.12	0.88 (0.44–1.77)	0.728
+	–	14 (12.85)	27 (25.71)	0.99; 0.41; 5.96	2.7 (1.22–5.99)	0.015
+	+	15 (13.76)	24 (22.86)	0.81; 0.41; 3.93	2.24 (1.01–4.97)	0.047

AD—Alzheimer’s dementia group, HC—healthy controls, APOEε4+—subjects with at least one ε4 allele, TNF*α* –850T—TT homozygotes + CT heterozygotes (dominant model), IL1A –889T—TT homozygotes + CT heterozygotes (dominant model), Ref.—reference group, β—coefficients beta; SE—standard error, OR—odds ratio, CI—confidence interval, significant OR and *p* value.
